# Discrepancies Between MDT Recommendations and AI-Generated Decisions in Gynecologic Oncology: A Retrospective Comparative Cohort Study

**DOI:** 10.3390/cancers18030452

**Published:** 2026-01-30

**Authors:** Vasilios Pergialiotis, Nikolaos Thomakos, Vasilios Lygizos, Maria Fanaki, Antonia Varthaliti, Dimitrios Efthymios Vlachos, Dimitrios Haidopoulos

**Affiliations:** First Department of Obstetrics and Gynecology, Division of Gynecologic Oncology, National and Kapodistrian University of Athens, 115 27 Athens, Greece; nthomakos@med.uoa.gr (N.T.); vaslyg@med.uoa.gr (V.L.); marfan@med.uoa.gr (M.F.); antoniavart@med.uoa.gr (A.V.); dgvlachos@med.uoa.gr (D.E.V.); dchaidop@med.uoa.gr (D.H.)

**Keywords:** artificial intelligence, ChatGPT, gynecological cancer, gynecologic oncology, multidisciplinary team

## Abstract

**Background:** Multidisciplinary tumor boards (MDTs) remain the foundation of gynecologic cancer management, yet increasing diagnostic complexity and rapidly evolving molecular classifications have intensified interest in artificial intelligence (AI) as a potential decision-support tool. This study aimed to evaluate the concordance between MDT-derived recommendations and those generated by ChatGPT 5.0 across a large, real-world cohort of gynecologic oncology cases. **Methods:** This single-center retrospective analysis included 599 consecutive patients with cervical, endometrial, ovarian, or vulvar cancer evaluated during MDT meetings over a 2-month period. Standardized anonymized case summaries were entered into ChatGPT 5.0, which was instructed to follow current ESGO guidelines. AI-generated staging and treatment recommendations were compared with MDT decisions. Discrepancies were independently assessed by two reviewers and stratified by malignancy type, disease stage, and treatment domain. **Results:** Overall concordance for FIGO staging was 77.0%, while treatment-related decisions demonstrated lower discordance, particularly in chemotherapy (8.2%) and targeted therapy (6.8%). The highest staging disagreement occurred in early-stage endometrial cancer (32.6%), reflecting the complexity of newly revised molecular classifications. In recurrent ovarian and cervical cancer, discrepancies were more pronounced in surgical and systemic therapy recommendations, suggesting limited AI capacity to integrate multimodal imaging, prior treatments, and individualized considerations. Vulvar cancer cases showed the highest overall agreement. **Conclusions:** ChatGPT 5.0 aligns with MDT decisions in many straightforward scenarios but falls short in complex or nuanced cases requiring contextual, multimodal, and patient-specific reasoning. These findings underscore the need for prospective, real-time evaluation, multimodal data integration, external validation, and explainable AI frameworks before LLMs can be safely incorporated into routine gynecologic oncology decision-making.

## Simple Summary

In this study, we compared AI-generated treatment recommendations with MDT decisions across 599 patients with cervical, endometrial, ovarian, and vulvar cancers. AI recommendations were generated using a structured, guideline-driven input format and evaluated across multiple decision domains, including staging, surgical management, and systemic therapy. Overall, concordance between AI and MDT recommendations was high, particularly in early-stage disease and in cancers with more standardized treatment pathways. However, discrepancies were more frequent in advanced and recurrent disease, with staging disagreements being the most common and often influencing downstream treatment recommendations. Discordance was especially notable in ovarian and endometrial cancer, reflecting the complexity of multimodal decision-making and the need to integrate imaging, molecular data, and prior treatments. These findings suggest that while AI tools may effectively support guideline-based decision-making in straightforward scenarios, their limitations become evident in complex cases requiring nuanced clinical judgment. Rather than replacing MDTs, AI systems may be best positioned as collaborative decision-support tools that enhance transparency and consistency while preserving clinician oversight in gynecologic oncology care.

## 1. Introduction

Multidisciplinary team (MDT) discussions are the backbone of modern gynecologic cancer management and ensure that diagnostic, staging, and treatment plans are based upon an integrated assessment of all relevant data [1]. Over the last two decades, there has been evidence of MDT discussions consistently leading to greater compliance with global treatment guidelines and improvements in cancer outcomes in cervix, endometrial, ovarian, and vulvar carcinoma. At the same time, there have been significant changes in current classifications defining all main gynecologic malignancies because of thorough revisions in the ESGO, ESTRO, and ESP treatment guidelines [2,3,4,5]. Consequently, the complexity level of data that needs to be processed during MDT discussions has increased profoundly, thereby triggering interest in technologies useful in these settings.

Artificial intelligence (AI) in the form of large language models (LLMs) has emerged as an attractive means to support clinical decision-making [6]. The newer versions of LLMs, including ChatGPT-4o and ChatGPT-5, show significant advances in terms of text comprehension and coherence in answers when applied to medical queries [7,8]. Emerging data from current studies suggest that advanced versions of LLMs can formulate relevant, accurate, and comprehensive answers to diverse medical queries related to any disease, at times equaling or outscoring answers provided by medical professionals in terms of accuracy, time taken, completeness, and empathy [9,10]. These qualities make it appear that advanced versions of LLMs can help in clinical practice, especially in settings needing rapid integration of guideline recommendations.

Although rapid progress has been made in the development of these capabilities, there has nevertheless been little direct transfer of these benefits to real-world oncologic management. To date, current research focuses solely on patient education exercises, simplified cases, or individual disease diagnostic procedure [11,12]. Very few of these studies have sought to measure the capability of these AI algorithms in precisely the same kind of clinical frameworks that exist in real-world treatment, meaning MDT frameworks [13,14]. Very few studies have explored whether AI outputs can compare or relate to the complex recommendations presented in these setups from experienced gynecologic oncologic teams with multiyear experience and many other health expert providers being part of the MDT [15,16,17]. These are important considerations because current MDTs are not merely predicated on guideline recommendations but also take into consideration issues like resectability and fertility issues, in addition to other issues, making personalized suggestions.

Additionally, there are some challenges that are uniquely presented in gynecologic cancers. First of all, the many cancers included under gynecologic cancers—cervical, endometrial, ovarian, and vulvar—have vastly different diagnostic and treatment strategies, all of which are described by different ESGO standards. Second, in many cases of gynecologic cancer, there are significant dependencies in surgery (e.g., primary cytoreduction, sentinel node application, parametrial involvement) that are hard to encode using written text alone. Finally, the complicated nature of classifications in these cancers (e.g., molecular risk factors in endometrial cancer, new prognoses in cervical cancer, changes in names and surgery classifications in ovarian cancer) makes it essential to constantly update these strategies. The assessment of whether the AI algorithms continue to work well in such a dynamic setting today constitutes an important precursor towards safely and effectively incorporating LLMs in cancer treatment.

The current analysis evaluates discrepancies among recommendations generated by AI algorithms under the ChatGPT 5.0 platform and MDT-driven decisions in real cases evaluated during the last 2 months in a tertiary referral center for the management of gynecological cancer in terms of surgery and/or use of adjuvant treatment in the form of chemotherapy, radiotherapy, and targeted/immune therapy, based on the latest ESGO guidelines. The purpose of the present study is to assess whether an advanced LLM can reproduce MDT-derived recommendations under real-world conditions and identify clinical contexts where discordance emerges. To accomplish this, we used a consecutive series of cervical, endometrial, ovarian, and vulvar cancers as they collectively represent the entire spectrum of gynecologic oncology practice. Evaluating AI-generated recommendations across these distinct cancer types allows assessment of whether large language models can adapt to heterogeneous decision-making environments rather than perform adequately only in linear, guideline-driven scenarios.

## 2. Materials and Methods

### 2.1. Study Design, Setting, and Ethical Approval

The present study was designed as a single-center, retroactive, observational study to examine the level of agreement between decisions reached by the Multidisciplinary Tumor Board and decisions proposed by AI in patients with gynecologic malignancies. The study was conducted in the First Department of Obstetrics and Gynecology, Alexandra General Hospital, National and Kapodistrian University of Athens, and involved a retrospective consecutive cohort of patients that were evaluated in our tumor board during the last 2 months. The study was approved by the ethics committee of the institution with the approval number 623/2025 and complied with the principles outlined in the Declaration of Helsinki, in 2023.

### 2.2. Study Population

There were 599 consecutive cases of MDT evaluation retrospectively assessed. The study considered eligible patients to be women with histologically proven cervical, endometrial, ovarian, or vulvar cancer, whose cases were presented formally in the context of consensus-reaching MDT meetings, while the cases not accompanied by complete information on imaging studies or final implementation of recommended management were excluded.

### 2.3. Data Collection and Variables

The data was harvested from patients’ Electronic Medical Records and multidisciplinary team documentation. The recorded variables included the following: demographic factors—age, performance status, and comorbidities; tumor factors—histological subtype, histological grading, and FIGO 202 stage; radiological evaluation and post-operative follow-up data; surgical information—extent of cytoreduction, resection status, and adjuvant therapy specifics. Recommendations were taken from official meeting minutes, which were signed by the chair of the MDT and the members in attendance. Every recommendation was the consensus of gynecologic oncologists, medical oncologists, radiation oncologists, radiologists, and pathologists.

### 2.4. AI Model and Input Standardization

ChatGPT-5.0 was used to make AI-driven treatment decisions in March 2025. For each patient, an anonymized version of the case summary, presented in the format of an internally standardized case summary template, was entered to the model. The input prompt included information on site, histology, stage, imaging characteristics, status of residuals after therapy, comorbidities, and prior therapies. The AI model was not trained nor fine-tuned on any institutional data sets. The model was programmed to offer treatment recommendations in accordance with the latest ESGO guidelines that are currently used as the reference standard for gynecological cancer management.

A fixed system prompt instructed the model to provide staging assessment and treatment recommendations strictly according to the most recent ESGO clinical practice guidelines applicable to gynecologic malignancies. The full prompt text and the structured case-input template are provided in Supplementary Material. Each case was submitted as a single-turn interaction. No follow-up questions, clarifications, or iterative dialogue were permitted. The AI session was reset between cases to prevent contextual carryover. No human intervention or modification occurred between prompt submission and output generation. MDT recommendations were documented prospectively in the institutional MDT report and served as the reference standard for comparative analysis. The process involved structured presentation and collective review of each patient’s radiological findings, pathology reports, and documented clinical history, including performance status, comorbidities, and prior treatments, with recommendations formulated in accordance with the latest ESGO clinical practice guidelines in each cancer field. Decisions were reached through multidisciplinary consensus prior to any treatment offered to patients and represent the standard-of-care clinical output rather than the reasoning of an individual clinician.

The output data included information on surgical management (extent, timing, and approach); systemic therapy (chemotherapy and/or immunotherapy); targeted or hormonal therapy; and follow-up or surveillance strategy.

The resulting recommendations were then exported and saved for comparative analysis.

### 2.5. Definition of Discrepancy and Review Procedure

Discrepancies between MDT and AI-generated recommendations were classified a priori as major or minor, based on their anticipated clinical impact. Major discrepancies were defined as differences that would alter the overall treatment strategy or therapeutic intent, including differences in decision-making concerning the primary treatment modality (e.g., surgery versus primary chemoradiation), omission or indication of systemic therapy, or differences likely to result in substantially different oncological outcomes. Variations in regimen selection, number of chemotherapy cycles (in the case of neoadjuvant or adjuvant chemotherapy) or follow-up strategy were considered as minor differences and were not recorded. Each decision domain (FIGO stage assignment, surgical management, systemic therapy, and targeted or hormonal therapy) was assessed independently. Concordance was defined as complete agreement between MDT and AI recommendations within a specific domain. A discrepancy was recorded only when a major discordance was present in that domain, as predefined. Disagreement in one decision domain did not imply disagreement in other domains.

Two reviewers, one of whom was a gynecologic oncologist and the other a data scientist, independently evaluated each pair of MDT–AI. The senior reviewer resolved ties. The reviewers were blind to patient identities and clinical results. The discrepancies were stratified by malignancy as cervical, endometrial, ovarian, or vulvar and by stage as early, advanced, or recurrent diseases, respectively.

### 2.6. Statistical Analysis

All statistical analyses were conducted with IBM SPSS Statistics version 29.0 (IBM Corp., Armonk, NY, USA). For numerical data, either mean values with standard deviations or medians with interquartile ranges were presented, whereas categorical data were presented as the number of cases and percentages. Discrepancy rates were contrasted between different malignancy types and areas of management with either chi-squared tests or Fisher exact tests, with further assessment of discrepancies according to malignancy stage and version of guidelines with post hoc tests. These were reported as proportions with 95% confidence intervals (CIs). Inter-rater agreement between MDT and AI was assessed using Cohen’s Kappa statistic with 95% CIs. Kappa was calculated for the FIGO stage among cases where both MDT and AI assigned a stage. The correlation between discrepancies in malignancy and management was explored with Spearman correlation tests wherein significance was defined at *p* < 0.05. Multivariable logistic regression was performed to identify predictors of discordance with discrepancy as the dependent variable and tumor type, early/advanced stage category (when available), recurrence status, and ECOG performance status as covariates. The results are presented as odds ratios (ORs) with 95% CIs. Statistical significance was defined as a two-sided *p* value < 0.05.

## 3. Results

A total of 599 patients with gynecologic malignancies were included in the analysis. Among these, 230 women were diagnosed with endometrial cancer, 228 with ovarian cancer, 100 with cervical cancer, and 4 with vulvar cancer. A total of 356 women were seen at the time of diagnosis, whereas the remaining 243 had disease relapse. Complete data were retrieved for all MDT-derived decisions and from the AI-generated process. Direct comparison of staging assignments was performed for all cases that had a primary disease. The decision of treatment was also compared on a case-by-case basis between MDT and AI recommendations.

The level of agreement among MDT-derived decisions and AI generated ones was higher when information about treatment recommendations was sought, whereas the assignment of stage was less concordant. Overall, MDT and AI agreed on FIGO staging in 462 of 599 cases (77.0%). For treatment decisions, discrepancies were less common. Specifically, the level of disagreement among the two was intermediate for the decision to proceed with surgery as well as with adjuvant treatment following surgery (0.5%), whereas it was lower for chemotherapy-related decisions (8.2%) and targeted therapy recommendations (6.8%) (Table 1).

Inferential analysis indicated that the inter-rater agreement between MDT and AI substantially differed in several treatment domains. Specifically, agreement was substantial for the decision of no further treatment (Kappa = 0.77, 95% CI 0.71–0.83), chemotherapy (Kappa = 0.73, 95% CI 0.67–0.78), and radiotherapy (Kappa = 0.79, 95% CI 0.73–0.85), whereas it was moderate for targeted/hormonal therapy (Kappa = 0.56, 95% CI 0.43–0.68) and surgical treatment decisions (Kappa = 0.64, 95% CI 0.55–0.71).

Following subgrouping of cases per cancer type, specific patterns were observed that seem to be directly related to the complexity of the staging systems. Specifically, endometrial cancer exhibited the highest rate of stage disagreement (32.6%), followed by cervical cancer (22.0%), while ovarian and vulvar cancers demonstrated lower rates (4.9% and 4.6%, respectively). Treatment-related discrepancy patterns also differed across malignancies, and the pattern seemed to be more influenced by the complexity of the procedures. Specifically, in ovarian cancer, disagreement occurred in 7.0% of cases in terms of surgical treatment and adjuvant treatment recommendations, representing the highest rates observed among all cancer types. In cervical cancer, significant discrepancies were observed in the decision to proceed with chemotherapy (4.0%) and targeted therapy (0.0%). Similar discordance was observed in chemotherapy-related recommendations among endometrial cancer cases, whereas rates of disagreement for surgical (5.7%) and adjuvant treatment (5.7%) were considerably lower. Discrepancies in vulvar cancer cases were moderate when in terms of the decision to proceed with surgery (9.8%) and the need to administer adjuvant treatment (9.8%), whereas discordance in terms of chemotherapy and or targeted therapy was extremely rare (Table 2).

**Figure 1:**
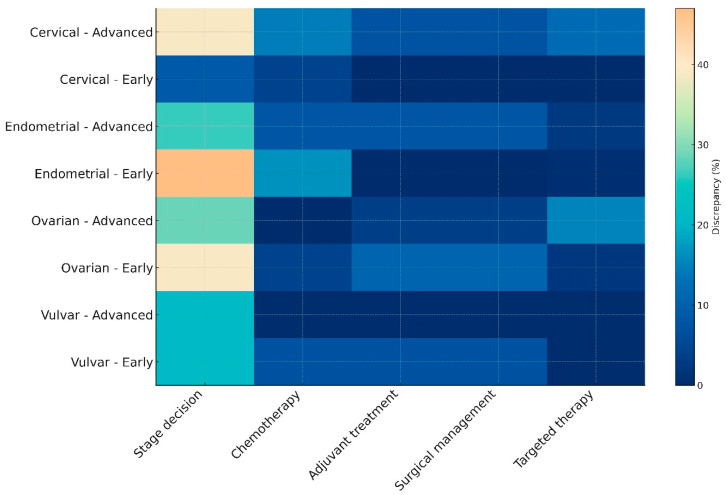
Heatmap illustrating discrepancy rates between MDT-driven and AI-generated decisions across cancer types and disease stages. This heatmap visualizes the percentage of discrepancies between ChatGPT 5.0 recommendations and multidisciplinary tumor board (MDT) decisions. Discrepancies are stratified by cancer type (cervical, endometrial, ovarian, or vulvar), disease stage (early or advanced), and decision domain (stage assignment, chemotherapy, adjuvant treatment, surgical management, or targeted therapy). Lighter colors denote higher disagreement, particularly notable in early-stage endometrial cancer and advanced ovarian cancer, whereas vulvar cancer demonstrates relatively lower discrepancy across domains.

Following subgrouping of cases to early or advanced disease, according to the latest FIGO staging systems we also observed staging assignment variations that seemed to influence treatment planning. As presented in the heatmap (Figure 1), the most notable differences among AI-generated decisions and MDT-driven proceedings were in the assignment of stage. Treatment-related decisions were less frequent, with vulvar cancer exhibiting the most notable agreement among the two entities. In the overall cohort, statistically meaningful differences among early- and advanced-stage cases were observed in chemotherapy and targeted therapy decisions, an observation that was mainly influenced by the results retrieved from cervical cancer cases (Table 3).

Multivariable logistic regression analysis indicated recurrent disease as an independent factor that was associated with a higher likelihood of MDT–AI treatment discrepancy (OR 2.79, 95% CI 1.52–5.12, *p* < 0.001). Tumor type, disease stage category, and performance status were not independently associated with discordance (Table 4).

## 4. Discussion

### 4.1. Principal Findings

The findings of our study suggest that AI tools cannot yet be considered trustworthy at the level of driving clinical decision-making in gynecologic oncology cases due to significant discrepancies with actual real-life practices. The most pronounced differences with actual MDT decisions were noted in clinical scenarios that required assessment with novel guidelines that include molecular profiling, namely early-stage endometrial cancer cases, denoting the complexity of current staging systems which is mainly based on the gross heterogeneity of early-stage clinical scenarios. It should be noted, however, that ChatGPT 5.0 aligned with the decisions of the MDT in the majority of clinical cases, therefore underscoring its potential future impact on clinical decision-making. Nevertheless, the cases in which the AI decision was in alignment with the actual human-driven recommendations were less complex and relatively straightforward.

### 4.2. Comparison with Existing Literature

The results of our analysis are consistent with the limited but growing body of evidence evaluating LLMs in gynecologic oncology decision-making. Specifically, Ebner et al. demonstrated that although ChatGPT could reproduce several core treatment principles in cervical cancer management, concordance with MDT decisions decreased when cases required individualized recommendations based on nuanced clinical or anatomical considerations [15]. Meyer et al. similarly reported that AI-powered tools perform well in generating guideline-consistent suggestions but fall short in scenarios requiring integration of multimodal data or interpretation of rapidly evolving classification systems, particularly those incorporating molecular stratification [16]. In a feasibility study, Levin et al. showed that ChatGPT could function as a supportive adjunct during MDT tumor board discussions but emphasized that the model lacked the depth and contextual reasoning necessary for autonomous clinical decision-making [17]. Compared with these studies, our findings are derived from a substantially larger and more diverse cohort and highlight that staging assignments constitute the domain with the highest discordance, especially in malignancies affected by recent, complex guideline updates such as endometrial cancer.

The comprehensive ability of AI, and in particular ChatGPT, has been previously evaluated in a study that distributed 804 questions encompassing four categories, namely true/false, multiple-choice, open-ended, and case-based scenarios [18]. Researchers observed the significant progression of AI in terms of accuracy, completeness, and guideline alignment and discussed that for all question types and complexity levels, ChatGPT-Omni consistently outperformed the earlier models and achieved near-perfect accuracy on easy and medium queries and higher scores on complex items. Despite this, however, they concluded that while Omni shows promise as a decision-support and educational tool, the variability across complexity levels raises concern, somewhat corroborating the findings of our study.

Recently, Rosati et al. published a systematic review summarizing evidence from AI language models built on natural language processing that were used in nine studies and also reached similar observations [19]. Specifically, researchers observed that while LMMs can be useful as adjunct tools, the evidence remains limited and heterogeneous to support them as independent clinical decision-makers. The main limitations in their ability to reach real-world clinical utility was their lower performance in complex or nuanced scenarios that required integration of multimodal information. Moreover, it remains unclear whether these models can correctly interpret imaging findings, surgical feasibility, comorbidities, or molecular classification data when these are either arbitrary or should be individualized outside standard staging decisions, supporting the idea that their reliability is highest only in straightforward, guideline-driven situations.

In our study, significant challenges in AI-directed decisions were observed in advanced case scenarios as well as recurrent disease, reflecting the inherent complexity of these cases. Staging disagreement remained the most prominent issue, and this discordance frequently carried downstream effects on treatment planning, particularly in recurrent disease where prior therapies, residual toxicities, and anatomical changes influence MDT recommendations in ways that are difficult to encode in text-based summaries. Specifically, in cases with recurrent and advanced ovarian cancer we observed the highest rates of discordance across both surgical and adjuvant treatment domains. These disagreements likely reflect the multifactorial nature of operability assessment, which depends not only on the pathology and radiological evidence of disease extent but also on anticipated surgical morbidity, patients’ performance status, platinum sensitivity, and possibly anticipated side-effects or complications. Such considerations are typically dynamically presented during MDT meetings and extend beyond guideline-defined decisions, hence reflecting the differences observed in our study. In cervical cancer, MDTs may integrate subtle variations in patterns of disease presentation and/or regression, as well as information about prior radiotherapy doses and distribution that may not be completely interpretable by the AI engine.

On the other hand, treatment-related discordance remained infrequent in early-stage disease, where guideline pathways are more linear and less dependent on individualized clinical nuance. Similarly, cases of vulvar cancer, irrespective of the actual stage, reflected a high level of concordance, indicating more standardized management algorithms and narrower therapeutic variability. Overall, while treatment-related discrepancies were less common than staging discrepancies across the whole cohort, they became more pronounced in advanced or relapsed disease, where multimodal decision-making is essential and guideline pathways are less linear. Importantly, vulvar cancer remained the exception, showing the highest concordance in advanced disease, likely owing to more standardized treatment algorithms.

The actual performance of AI-drive decisions in gynecologic oncology remains unexplored to date. Data from other forms of cancer are conflicting. Specifically, in a prospective cohort study conducted on 250 patients with colorectal cancer from China, researchers observed that AI-driven decisions had a small discordance from those of the MDT meeting, with patient age, cancer stage, and the consideration of previous therapy details being the main factors that influenced different approaches in decision-making [20]. In more complex scenarios, involving decisions from sarcoma tumor boards, the performance of AI decisions and in particular of ChatGPT 4.0 was found to be inferior, particularly when individualization of cases was considered essential in the presence of complex imaging and pathology results or induction of patients in clinical trials and or prior systemic therapies [21].

### 4.3. Strengths and Limitations

The main strength of our study is the large, consecutive, real-world cohort used, encompassing 599 women with all major forms of gynecologic cancer including patients with both primary and recurrent disease. This sample size allowed for a robust comparison between MDT-driven decisions and those generated by an advanced LLM, which was evaluated in a manner that closely reflects the potential use that the LLM would gain in actual clinical practice. Furthermore, the use of a standardized method of AI input preparation and a structured discrepancy assessment performed independently by two reviewers ensures consistency, together minimizing subjective interpretation of findings. The investigation of the most important distinct domains of clinical decision-making, including staging, need for surgical management, systemic therapy, and targeted/immune treatment, provided a thorough analysis able to detect cancer-specific and stage-specific patterns of discordance. This permitted an in-depth evaluation that revealed the difficulties that the model faced in complex cases, including early-stage endometrial cancer and recurrent ovarian cancer.

Nevertheless, several limitations should be acknowledged. Firstly, AI recommendations were based solely on structured textual case summaries. Therefore, integration of specific data, such as raw radiological imaging data, as well as subjective clinical judgement regarding treatment options in patients with multiple comorbidities and physical capacity-related problems might not be considered by the algorithm. Moreover, limitations in institutional resources as well as patient preferences could not have been considered. Therefore, one could speculate that fine-tuning might be essential in complex cases to increase the accuracy of the model and better reflect the priorities and reasoning of the MDT tumor board; ultimately, supporting this development may accurately the capture the actual tone and wishes of the MDT tumor board in order to establish a contextually accurate therapeutic plan. It is also worth noting that despite the fact that the model was instructed to follow the latest ESGO guidelines, the actual algorithm and the internal training processes used to reach it in each case remain obscure; therefore, the possibility of incomplete assimilation of recent guideline updates cannot be excluded. Another potential issue of this study is the lack of external validation of the actual recommendations of the MDT tumor board of our institution. Despite the fact that we considered the decisions made as the reference standard, MDT meeting proceedings are never perfect as differences between institutions may influence how reproducible these comparisons are [22].

### 4.4. Implications for Clinical Practice

The results indicate that while advanced LLMs, such as ChatGPT 5.0 as used in this study, can reproduce a substantial proportion of MDT-derived decisions, their reliability varies considerably depending on the disease complexity, availability of multimodal information, and need for individualized clinical judgment. Prior work in medical decision support suggests that collaborative frameworks may improve transparency, consistency, and guideline adherence while preserving clinician oversight and accountability. Specifically, in a recent review of the clinical evidence, Liu et al. suggested that human–AI teams in healthcare combine clinician expertise with AI assistance and produce outcomes greater than either alone, emphasizing the need to understand not just whether AI agrees with experts but how clinicians and AI interact as collaborative partners in complex decision contexts [23]. Empirical work on AI-augmented co-design in healthcare demonstrates that AI tools can help to quantify teamwork behaviors and collective intelligence outcomes, in order to improve participation dynamics, balance contributions, and provide structured reflection in collaborative environments [24]. This underlines the need for future research targeting mechanisms that could help to augment multidisciplinary clinical interactions with the use of AI rather than replace them. On the other hand, a systematic review focusing on the use of AI in clinical decision-support systems denoted that transparency and interpretability are critical elements to help establish clinician trust, accountability, and safe integration [25]. However, given the absence of focused studies and the existing gaps, several barriers persist that still seem to prohibit their adoption in clinical care. In this context, the longer-term goal of AI integration in MDT settings should be the development of collaborative frameworks that explicitly support uncertainty communication, preserve clinician accountability, and mitigate risks of automation bias or over-reliance. In their current form, AI tools may serve as supportive resources to summarize guideline-based pathways or potentially aid in preliminary case preparation. The results of the current research indicate, however, that they cannot replace multidisciplinary deliberation as this relies on several factors that supersede the actual context of retrieved patient files. Particular caution is warranted in early-stage endometrial cancer and recurrent ovarian cancer cases, where staging intricacies and operability assessments demand nuanced interpretation beyond the AI’s present capability that is based merely in guideline-driven decision algorithms.

Therefore, the findings of this study support a model of expert–machine collaboration, in which AI systems function as decision-support tools that augment, rather than replace, multidisciplinary expertise. In such frameworks, AI may assist by standardizing guideline-based recommendations or highlighting potential management options, while final decisions remain under clinician oversight.

Future research should focus on dynamic assessment of AI tools, integrating them into real-time MDT meetings, ideally with the use of voice-recognition-based interfaces which could offer valuable insight into how these systems respond dynamically to clinical debate and iterative decision-making. The introduction of structured models of expert–machine collaboration within MDT settings could help to evaluate how clinicians interact with AI-generated outputs, interpret uncertainty, and integrate algorithmic suggestions into collective decision-making processes. It could also help to verify whether such processes enhance physician confidence by increasing transparency in guideline application, or whether they introduce additional cognitive burden and stress in cases that require nuanced and individualized clinical judgment. In parallel with these efforts, it would also be useful to consider formal approaches to model uncertainty in MDT–AI concordance, using multi-criteria group-decision-making frameworks. Such methodologies could allow concordance and discordance to be expressed through interval-based or probabilistic representations rather than binary classifications, better reflecting the inherent variability and subjectivity of complex oncologic decision-making. Incorporating uncertainty into comparative analyses may offer a more nuanced interpretation of agreement between AI-driven and MDT-derived recommendations, particularly in clinical scenarios where multiple guideline-acceptable options coexist or where expert judgment diverges due to patient-specific factors. Another point that is worthy of investigation is the actual validity of multimodal data inputs, including radiologic imaging with image recognition capabilities; the use of pathology slides would help to overcome current limitations associated with text-only case summaries. This way, the AI-driven algorithm would incorporate in its system these modalities and would thoroughly interpret the same spectrum of information that is routinely evaluated by individual MDT members. External validation across multiple institutions and MDT boards is essential to evaluate if discrepancies among AI-driven recommendations are actually the result of a divergence from guidelines that is owed to institutional resources, physician competencies, or patient-specific factors. This way, we will better understand if the difficulties in reaching unanimous decisions are based on the actual limitations of the AI algorithm or can be considered inherent due to facility/patient-based restrictions. Explainable frameworks are also needed to enable physicians to understand AI reasoning behind output recommendations. This way, objective assessment of the algorithm`s performance will be available. Moreover, it would be useful to use prompt-engineering and ablation approaches to determine whether AI discordance arises from limitations in reasoning, incomplete contextual inference, or sensitivity to prompt structure. Such analyses would help to clarify which components of the decision-making process are most vulnerable to error and inform the development of more robust, transparent, and clinically reliable AI-assisted decision-support systems. Lastly, when our knowledge of AI reasoning becomes more comprehensive, one might consider conducting longitudinal studies to evaluate the impact of AI-driven decisions on patient clinical outcomes (including survival outcomes and perioperative endpoints), thus enabling an active comparison with current MDT-meeting practices.

## 5. Conclusions

In this large, real-world cohort, ChatGPT 5.0 demonstrated a moderate level of agreement with MDT-derived decisions. Concordance between AI-driven decisions and MDT recommendations was manifested mainly in straightforward clinical scenarios and guideline-driven treatment pathways. However, discrepancies emerged in cases requiring complex staging assessments, individualized surgical judgement, and integration of multimodal information that underline the limitations of AI tools in clinical decision-making in real practice. These findings indicate that while AI tools may support preliminary case evaluation, and potentially suggest a preliminary care plan, they cannot yet fully replicate the nuanced reasoning, contextual interpretation, and multidisciplinary expertise that guide contemporary gynecologic oncology care. To fully realize the clinical potential of AI in gynecologic oncology, future research must incorporate dynamic, multimodal, and externally validated approaches that capture the complexity of real MDT decision-making. As AI systems become more transparent and better aligned with imaging, pathology, and real-time clinical dialogue, their impact on patient outcomes can be rigorously evaluated and meaningfully compared with current MDT-driven standards to help improve patient care for gynecological cancer patients.

## Figures and Tables

**Table 1:** Overall discrepancy rates between MDT-derived and AI-generated decisions across all gynecologic cancer cases. Summary of concordance and discordance percentages between MDT decisions and AI recommendations for staging, surgical treatment, further/adjuvant treatment, chemotherapy, and targeted therapy among 599 patients. Concordant proportions represent the complement of the corresponding domain-specific discrepancy rates, with concordance and discordance assessed independently for each decision domain.

Variable	N	Concordant (n, %)	Discrepant (n, %)
Stage assignment (AI vs. MDT)	599	462 (77.1%)	137 (22.9%)
Further treatment decision	599	536 (89.5%)	63 (10.5%)
Surgical treatment decision	599	536 (89.5%)	63 (10.5%)
Chemotherapy recommendation	599	550 (91.8%)	49 (8.2%)
Targeted therapy recommendation	599	558 (93.2%)	41 (6.8%)

**Table 2:** Discrepancy rates between MDT and AI decisions stratified by cancer type. Data suggest discordance proportions for staging, surgical decisions, adjuvant treatment, chemotherapy, and targeted therapy across cervical, endometrial, ovarian, and vulvar malignancies. Significant variability is observed between tumor types, with the highest staging discrepancies noted in endometrial cancer and the highest treatment-related discrepancies in ovarian cancer.

Cancer Type	N	Stage Discrepancy (n, %)	Further Tx Discr.	Surgical Tx Discr.	Chemo Discr.	Targeted Tx Discr.
Cervical	100	22 (22.0%)	7 (7.0%)	7 (7.0%)	14 (14.0%)	11 (11.0%)
Endometrial	230	75 (32.6%)	13 (5.7%)	13 (5.7%)	29 (12.6%)	10 (4.3%)
Ovarian	228	34 (14.9%)	39 (17.1%)	39 (17.1%)	4 (1.8%)	20 (8.8%)
Vulvar	41	6 (14.6%)	4 (9.8%)	4 (9.8%)	2 (4.9%)	0 (0.0%)
*p*-value		<0.001	<0.001	<0.001	<0.001	0.026

**Table 3:** Discrepancy rates between MDT and AI decisions stratified by cancer type and disease stage (early vs. advanced). Differences in concordance across treatment domains (surgery, chemotherapy, targeted therapy, or further treatment) and staging accuracy when cases are subgrouped by both malignancy and disease stage. Notable trends include increased chemotherapy and targeted therapy discrepancies in advanced-stage cervical cancer and higher staging divergence in early-stage endometrial cancer.

Cancer Type	Discrepancy Variable	Early Stage n/N (%)	Advanced Stage n/N (%)	*p*-Value
Overall cohort (n = 367)	Further treatment	6/215 (2.8%)	8/146 (5.5%)	0.131
	Surgical treatment	6/215 (2.8%)	8/146 (5.5%)	0.131
	Chemotherapy	26/215 (12.1%)	9/146 (6.2%)	0.001
	Targeted therapy	2/215 (0.9%)	14/146 (9.6%)	<0.001
	Stage discrepancy	85/215 (39.5%)	44/146 (30.1%)	0.117
Cervical cancer	Further treatment	0/23 (0.0%)	3/41 (7.3%)	0.232
	Surgical treatment	0/23	3/41	0.232
	Chemotherapy	1/23 (4.3%)	6/41 (14.6%)	0.017
	Targeted therapy	0/23	5/41 (12.2%)	0.003
	Stage discrepancy	2/23 (8.7%)	16/41 (39.0%)	0.027
Endometrial cancer	Further treatment	0/132 (0.0%)	3/38 (7.9%)	0.001
	Surgical treatment	0/132	3/38	0.001
	Chemotherapy	22/132 (16.7%)	3/38 (7.9%)	0.179
	Targeted therapy	1/132 (0.8%)	1/38 (2.6%)	0.345
	Stage discrepancy	62/132 (47.0%)	10/38 (26.3%)	0.023
Ovarian cancer	Further treatment	5/46 (10.9%)	2/53 (3.8%)	0.170
	Surgical treatment	5/46	2/53	0.170
	Chemotherapy	2/46 (4.3%)	0/53 (0.0%)	0.125
	Targeted therapy	1/46 (2.2%)	8/53 (15.1%)	0.026
	Stage discrepancy	18/46 (39.1%)	15/53 (28.3%)	0.254
Vulvar cancer	Further treatment	1/14 (7.1%)	0/14 (0.0%)	0.309
	Surgical treatment	1/14	0/14	0.309
	Chemotherapy	1/14	0/14	0.309
	Targeted therapy	—	—	N/A
	Stage discrepancy	3/14 (21.4%)	3/14 (21.4%)	1.000

**Table 4:** Multivariable logistic regression analysis of predictors of MDT–AI treatment discrepancy. The dependent variable was the presence of at least one discordant treatment recommendation (further treatment, surgical management, systemic therapy, or targeted/hormonal therapy). Odds ratios (ORs) are presented with 95% confidence intervals (CIs).

Variable	Odds Ratio (OR)	95% Confidence Interval	*p* Value
Tumor type			
Cervical cancer	Reference	–	–
Endometrial cancer	0.81	0.45–1.44	0.48
Ovarian cancer	1.29	0.73–2.28	0.38
Vulvar cancer	0.94	0.36–2.44	0.90
Disease setting			
Primary disease	Reference	–	–
Recurrent disease	2.79	1.52–5.12	<0.001
Disease stage			
Early stage	Reference	–	–
Advanced stage	1.42	0.88–2.31	0.15
Performance status			
ECOG 0–1	Reference	–	–
ECOG ≥ 2	1.31	0.62–2.76	0.48

## Data Availability

The data supporting the findings of this study are available on reasonable request from the corresponding author. Restrictions apply due to patient confidentiality.

## Supplementary Materials

The following supporting information can be downloaded at https://www.mdpi.com/article/10.3390/cancers18030452/s1.
