# Supplementary material for: Discrepancies Between MDT Recommendations and AI-Generated Decisions in Gynecologic Oncology: A Retrospective Comparative Cohort Study
Source: Cancers (Basel). 2026 Jan 30;18(3):452. doi: 10.3390/cancers18030452 (PMC12897028; doi:10.3390/cancers18030452)
Supplement: Supplementary file 1 [file cancers-18-00452-s001.zip › cancers-4093821-supplementary.pdf]

System prompt (verbatim)

You are an AI clinical decision support system assisting in the management of gynecologic oncology patients. Your task is to independently determine the FIGO stage and provide evidence-based treatment recommendations strictly in accordance with the most recent ESGO clinical practice guidelines applicable to the specified malignancy. The FIGO stage has not been provided and must be inferred solely from the clinical, imaging, and pathology report information contained in the case summary.

Base your recommendations only on the information provided. Do not introduce assumptions or external data. If information is insufficient to assign a FIGO stage or recommend treatment, explicitly state this.

For each case, provide output in the following four domains:

(1) FIGO stage determination

(2) Surgical management (timing, extent, and approach)

(3) Systemic therapy (chemotherapy and/or immunotherapy)

(4) Targeted or hormonal therapy (if applicable)

(5) Follow-up or surveillance strategy

Structured Case-Input Template

Each case was submitted using the following standardized anonymized input format. The MDT-assigned FIGO stage was deliberately excluded to let ChatGPT 5.0 determine it and provide therapeutic decisions. Similarly, MDT conclusions, or treatment recommendation were not included in the input. All cases were submitted as single-turn interactions - no multi-turn dialogue, clarification prompts, or iterative corrections were permitted.

Patient age:

Tumor site: (cervical / endometrial / ovarian / vulvar)

Histology:

Tumor grade (if applicable):

Imaging findings: (summary of CT/MRI/PET findings relevant to local extent, nodal involvement, and metastases)

Pathological findings: (if biopsy or surgery performed)

Molecular profile: (e.g. POLE, MMR, p53; if available)

Residual disease status: (if prior surgery performed)

Recurrence status: (primary / recurrent)

Prior treatments: (surgery, chemotherapy, radiotherapy, targeted therapy)

Relevant comorbidities:

Factors that were predetermined as minimum inputs that were needed to be evaluated per cancer type

Endometrial Cancer

Pathological parameters

Histological subtype (endometrioid, serous, clear cell, carcinosarcoma)
Tumor grade (where applicable)
Depth of myometrial invasion (if biopsy or surgical specimen available)
Lymphovascular space invasion (when reported)
Molecular classification according to ESGO/WHO recommendations:
POLE mutation status
Mismatch repair (MMR) status
p53 expression pattern

Imaging techniques

Pelvic MRI (primary modality):

- Myometrial invasion depth
- Cervical stromal involvement
- Adnexal or serosal extension

CT chest/abdomen/pelvis:

- Nodal involvement
- Distant metastases

PET-CT (when available):

- Nodal or extra-pelvic disease assessment

Ovarian, Fallopian Tube, and Primary Peritoneal Cancer

Pathological parameters

- Histological subtype (high-grade serous, low-grade serous, endometrioid, mucinous, clear cell)
- Tumor grade
- BRCA1/2 germline or somatic mutation status (if available)
- Homologous recombination deficiency (HRD) status (when available)
- Cytology or biopsy confirmation (omentum, peritoneum)

Imaging techniques

- Contrast-enhanced CT chest/abdomen/pelvis (primary modality):
  - Disease distribution
  - Peritoneal carcinomatosis
  - Ascites
  - Nodal involvement
- MRI abdomen/pelvis:
  - Local pelvic disease characterization
- PET-CT:
  - Indeterminate nodal or distant disease

Cervical Cancer

Pathological parameters

- Histological subtype (squamous cell carcinoma, adenocarcinoma, adenosquamous)
- Tumor differentiation grade
- Lymphovascular space invasion
- HPV prevalence and typing

Imaging techniques

- Pelvic MRI (primary modality):
  - Tumor size
  - Parametrial invasion
  - Vaginal or pelvic wall involvement
- PET-CT:
  - Pelvic and para-aortic nodal involvement
  - Distant metastatic disease
- CT chest/abdomen:
  - Evaluation of extra-pelvic spread when PET-CT unavailable

Vulvar Cancer

Pathological parameters

- Histological subtype (squamous cell carcinoma variants)
- Tumor size
- Depth of invasion
- Margin status (if excisional biopsy performed)
- Lymphovascular space invasion

Imaging techniques

- Pelvic MRI:
    - Local tumor extent
    - Involvement of urethra, vagina, or anus
- Ultrasound or CT:
    - Inguinal and femoral lymph node assessment
- PET-CT (selected cases):
    - Nodal or distant disease evaluation

Multidisciplinary Tumor Board composition

The multidisciplinary tumor board (MDT) consisted of board-certified

- gynecologic oncologists,
- medical oncologists,
- radiation oncologists,
- pathologists,

all of them had subspecialty experience in gynecologic malignancies expanding to a minimum of 5 years. Each year approximately 500 patients are referred for treatment in our institution.

MDT meetings are held on a weekly basis upon formal standardized presentation. Clinical recommendations are reached by consensus agreement among MDT members. In cases of disagreement in initial decisions, discussion continues until consensus is achieved. No formal majority-vote decisions are recorded. MDT recommendations were documented prospectively in the institutional MDT report and served as the reference standard for comparative analysis.
